# Transcription Factor AtOFP1 Involved in ABA-Mediated Seed Germination and Root Growth through Modulation of ROS Homeostasis in *Arabidopsis*

**DOI:** 10.3390/ijms23137427

**Published:** 2022-07-04

**Authors:** Hemeng Wang, Dongrui Zhang, Xi’nan Zhou, Ganghua Zhou, Wenbo Zong, Lingling Chen, Ying Chang, Xiaoxia Wu

**Affiliations:** 1 College of Life Sciences, Northeast Agricultural University, Harbin 150030, China; hemengwang@yeah.net (H.W.); geraldtwinersom@gmail.com (D.Z.); 2 College of Agriculture, Northeast Agricultural University, Harbin 150030, China; 3 Biotechnology Research Institute, Chinese Academy of Agricultural Sciences, Beijing 100081, China; xinan15245116526@163.com; 4 Key Laboratory of Molecular Epigenetics of MOE, Institute of Genetics and Cytology, Northeast Normal University, Changchun 130024, China; zhough767@nenu.edu.cn; 5 Department of Biochemistry and Molecular Biology, College of Basic Medical Science, Jilin University, Changchun 130021, China; zwenbo523@163.com; 6 MOA Key Laboratory of Crop Ecophysiology and Farming System in the Middle Reaches of the Yangtze River, College of Plant Science & Technology, Huazhong Agricultural University, Wuhan 430070, China; chenlingling1926@gmail.com

**Keywords:** OFP transcription factor, protein interaction, CHIP-SEQ, abscisic acid, reactive oxygen species

## Abstract

Ovate family proteins (OFPs) are valued as a family of transcription factors that are unique to plants, and they play a pluripotent regulatory role in plant growth and development, including secondary-cell-wall synthesis, DNA repair, gibberellin synthesis, and other biological processes, via their interaction with TALE family proteins. In this study, CHIP-SEQ was used to detect the potential target genes of AtOFP1 and its signal-regulation pathways. On the other hand, Y2H and BIFC were employed to prove that AtOFP1 can participate in ABA signal transduction by interacting with one of the TALE family protein called AtKNAT3. ABA response genes are not only significantly upregulated in the *35S::HAOFP1* OE line, but they also show hypersensitivity to ABA in terms of seed germination and early seedling root elongation. In addition, the AtOFP1-regulated target genes are mainly mitochondrial membranes that are involved in the oxidative–phosphorylation pathway. Further qRT-PCR results showed that the inefficient splicing of the respiratory complex I subunit genes *NAD4* and *NAD7* may lead to ROS accumulation in *35S::HA-AtOFP1* OE lines. In conclusion, we speculated that the overexpression of AtOFP1 may cause the ABA hypersensitivity response by increasing the intracellular ROS content generated from damage to the intima systems of mitochondria.

## 1. Introduction

Abscisic acid (ABA), a well-known stress hormone, affects various processes of plant growth and development and environmental adaptation, such as seed germination, root development, stomatal movement, and stress tolerance [1,2,3]. The central ABA signaling transduction pathway initially relies on the pyrabactin resistance 1 (PYR1) and PYR1-like (PYL) receptors to perceive ABA and inhibit type 2C protein phosphatases (PP2Cs). The inactivation of PP2Cs results in the phosphorylation and activation of sucrose nonfermenting 1 (Snf1)-related protein kinase 2 (SnRK2) family members. These activated SnRK2s then regulate downstream transcription factors (TFs) through phosphorylation, such as ABA-responsive element (ABRE)-binding factors (ABFs) and ABA-insensitive 5 (ABI5), which, in turn, stimulate the expression of ABA-responsive genes [4,5]. TFs that are involved in ABA signal transduction contain specific cis-acting elements in their promoter regions. For example, *RD29A* and *RD29B*, which belong to the AP2 family, play a key role in responding to ABA signaling and resistance to stress [6]. Another AP2 family member, *ABI4*, is also involved in the regulation of the ABA inhibition of seed germination [7]. ABREs are typically conserved in the promoter regions of ABA-responsive genes, and the bZIP TF tends to positively mediate seed dormancy and seedling development by binding ABREs [8]. When the bZIP-type TFs ABF3 and ABF4 are overexpressed in *Arabidopsis*, they are hypersensitive to ABA in both seed germination and seedling growth [9]. *ABI5* (abscisic-acid-insensitive 5) binds to ABREs during abiotic stress and is strongly induced by exogenous ABA. Moreover, ABI5 also negatively regulates the expressions of the *Em1* and *Em6* genes that encode late embryogenesis abundant (LEA) proteins, which thus affects the seed germination and subsequent growth [10]. In addition, the MYC and MYB transcription factors within the bHLH family are also involved in the responses of plants to ABA and drought stress [11]. The overexpression of *AtMYC2* and *AtMYB2* in *Arabidopsis* lead to an ABA-hypersensitive phenotype, which enhances their tolerance to stress and accelerates the expression of dehydration-responsive protein 22 (*RD22*) in plants [12].

Moreover, ROS partially participate in the ABA-signaling transduction pathway as second messengers [13,14]. When ROS such as hydrogen peroxide (H_2_O_2_) and superoxide (O_2_^−^) over-accumulate, plants tend to generate a wide range of antioxidants (SOD, POD, and CAT) to scavenge the cytotoxic substances that they cause [15,16]. Typically, the dysfunction of the mitochondrial electron transport chain (ETC) can lead to redox imbalance and ROS accumulation. Under normal circumstances, electrons consecutively pass through four complexes (from complex I to complex IV) and are eventually delivered to oxygen in the form of water. However, if the respiratory chain cannot transfer electrons to the downstream coenzyme (Q), the leaked electrons directly combine with oxygen to form many superoxide anions (O_2_^-^), which are further oxidized to produce H_2_O_2_ and OH^−^ [17]. According to the existing studies, mitochondria genes in *Arabidopsis* contain multiple introns. Whereas *Arabidopsis* ETC complex I NADH dehydrogenase subunit 1 (*NAD1*), *NAD2*, and *NAD5* contain four introns, *NAD4* contains three, *NAD7* contains two, and *RPL2* (ribosomal protein large subunit 2), *RPS3* (ribosomal protein small subunit 3), *ccmFc* (cytochrome c maturation subunit F C-terminus), and *COX2* (cytochrome c oxidase subunit 2) each contain one [18]. Therefore, mutants with abnormal intron splicing cause a variety of severe phenotypes, including seedling growth retardation, reduced fertility, and abiotic stress responses [19]. For instance, the loss of mitochondrial stabilization factor (*MTSF1*) in *Arabidopsis* means that it cannot obtain a normal splice of the *NAD4* gene, which directly results in the formation of a shriveled seed morphology. The splicing failure of the first intron of the *NAD1* gene similarly leads to slow growth, smaller seeds, and curly leaves [20,21]. In terms of the *NAD2* gene in the maize mitochondrial genome, if its fourth intron is not splicing correctly, then it also produces seeds with an atrophic form [22].

As a plant-specific transcriptional regulatory protein family, ovate family proteins (AtOFPs) are essential for multiple aspects of plant growth and development in various plants, including meristem function and maintenance, lateral-organ specification, and leaf development and branching [23,24,25]. AtOFPs harbor a 70 aa conserved domain in the C- of the coding sequences that is known as the OVATE, or unknown 623 [26]. The absence of predicted DNA-binding domains in AtOFPs promotes their interaction with other proteins to regulate the downstream target genes. In 2005, the large-scale Y2H preliminarily identified 13 three-amino-acid loop extension (TALE) homeodomain transcription factors containing the KNOX and BELL classes that interact with AtOFP1 [27]. Given the importance of OFP1–TALE protein complexes, a series of interactions were successively verified on this basis. Early reports demonstrated that AtOFP1 can form a weak connection with AtKNAT7 to jointly regulate the synthesis of the plant secondary cell wall [25]. Our laboratory found that AtOFP1 not only interacts with AtBLH3, but also reduces its transcriptional activity, which alters the plant transition process from vegetative growth to reproductive growth [28]. AtOFP1 and AtATH1 mainly interact through the HD rather than the SKY-BELL domain, and this affects stem elongation and the fusion of the flower-base boundary [29]. However, we do not know whether AtOFP1 interacts with other TALE proteins.

In this study, we found that AtOFP1 participates in the ABA response by interacting with the TALE member KNAT3. Furthermore, according to the ChIP-SEQ and qRT-PCR results, the potential downstream genes mediated by AtOFP1 are mainly mitochondrial electron transport chain (ETC)-complex subunit genes, and AtOFP1 likely alters the inner ROS content by regulating the splicing of mitochondrial Complex I *NAD4* intron 3, as well as *NAD7* intron 2. The additional GSH partially rescues the ABA sensitivity in the *35S::HAOFP1* OE line, which is due to ROS accumulation.

## 2. Results

### 2.1. AtOFP1 Can Physically Interact with AtKNAT3

**Figure 1:**
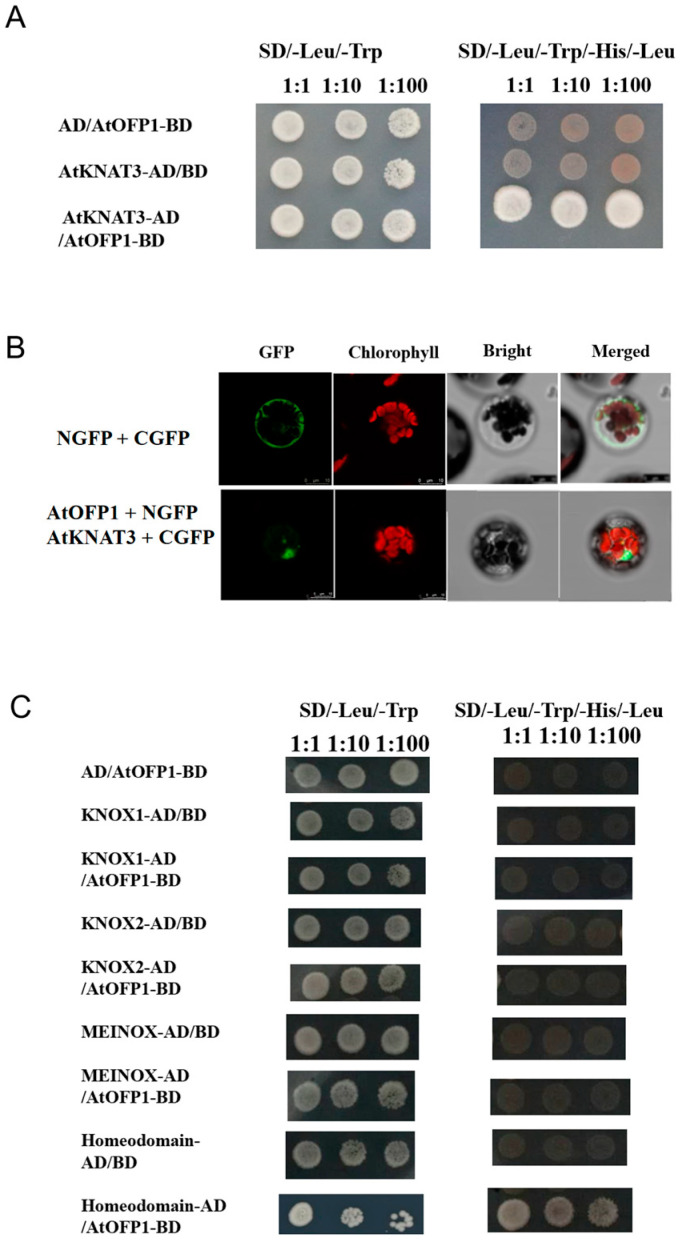
Protein interaction between AtOFP1 and AtKNAT3. (**A**)The full-length AtOFP1 and AtKNAT3 sequences were cloned from *Arabidopsis* and fused to the pGBKT7 and pGADT7 vectors, respectively; pGBKT7 (BD) and pGADT7 (AD)-AtKNAT3 were used as negative controls, while pGBKT7–AtOFP1 and AD were applied for self-activation activity. The yeast cells were selected on SD medium without Trp (tryptophan) and Leu (leucine), then the interaction relationship was measured by growing them on selective SD medium lacking Trp, Leu, Ade (adenine), and His (histidine). (**B**) The BiFC images were analyzed by a confocal microscope. Top: images of co-transfection of NGFP and CGFP were regarded as negative control. Bottom: co-expression of AtKNAT3–CGFP + AtOFP1–NGFP in *Arabidopsis* protoplasts. The green fluorescence revealed protein interaction between AtKNAT3 and OFP1. Bars = 10 μm. (**C**) The structure fragments of AtKNAT3, including KNOX1, KNOX2, MEINOX (KNOX1 + KNOX2), and HD domains, were cloned and inserted into pGADT7, separately. Then, different combinations between truncated fragments and pGBKT7-AtOFP1 were harbored in yeast AH109 cells.

To explore whether one of the TALE proteins, AtKNAT3, can interact with AtOFP1, we used a yeast two-hybrid (Y2H) screening system to co-transform the pGADT7–AtKNAT3 and pGBKT7–AtOFP1 yeast expression vectors into AH109 yeast cells. As shown in Figure 1A, whereas all three combinations (BD-AtOFP1 and AD-AtKNAT3; BD-AtOFP1 and AD; BD and AD-AtKNAT3) grew well in the SD-Leu/-Trp 2-defective medium, only the combinations of the AtOFP1 DNA-binding domain (DBD) and AtKNAT3 activation domain (AD) fusion proteins displayed normal yeast growth in SD/-Leu/-Trp/-His/-Ade 4 deficient screening medium, which means that AtOFP1 can interact with AtKNAT3 in vitro. In this study, we tested the combination of pGBKT7–AtOFP1 and AD-empty protein to exclude the possibility of self-activation activity in pGBKT7–AtOFP1. Furthermore, according to the bimolecular fluorescence complementation (BiFC), the coexpression of GFPC–AtKNAT3 and GFPN–AtOFP1 in the *Arabidopsis* protoplast could produce green fluorescence, which suggests that AtOFP1 and AtKNAT3 also physically interact in vivo (Figure 1B).

In addition, AtKNAT3 is composed of the KNOX1, KNOX2, and HD domains [30] (Figure S1). To confirm the specific domain that interacts with AtOFP1, we fused four truncated fragments of AtKNAT3, including the KNOX1, KNOX2, MEINOX (KNOX1 + KNOX2), and HD domains, with pGADT7. According to the results, the HD domain within AtKNAT3 can interact with AtOFP1, which is necessary for the formation of the AtOFP1–AtKNAT3 protein complex (Figure 1C) (Figure S2).

According to these results, AtKNAT3 is a putative interacting partner of AtOFP1, and AtOFP1 physically interacts with the HD domain of AtKNAT3.

### 2.2. AtOFP1 Influences Expression Patterns of ABA-Pathway-Related Genes

**Figure 2:**
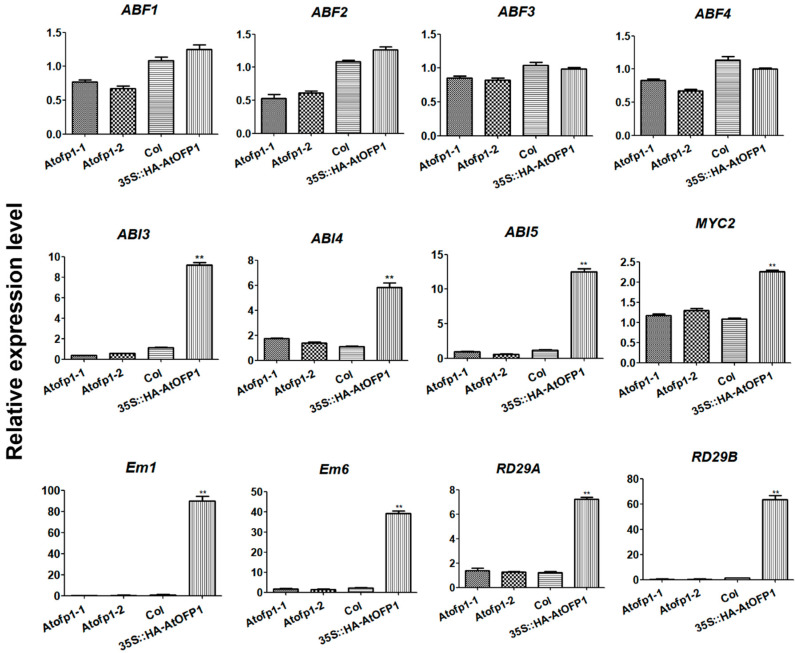
Expression levels of ABA-responsive genes. Note: qRT-PCR analysis of ABA-responsive genes in 10-day-old seedlings of Col, *Atofp1-1*, *Atofp1-2* and *35S::HA-AtOFP1*. Transcript levels of these genes were quantified relative to that of Actin2/8. Each data bar represents the means ± SE (*n* = 3). Asterisk (**) indicates significantly statistical difference (*p* < 0.01).

According to the evidence, AtKNAT3 participates in the ABA-mediated response during early seedling development [31]. To investigate the potential involvement of AtOFP1 under ABA treatment, we further examined the expressions of ABA-signaling-related genes in Col-0, *Atofp1-1*, and *Atofp1-2* mutants and the *35S::HAOFP1* OE line. Among them, *ABF1*, *ABF2*, *ABF3*, *MYC2*, *ABI3*, and *ABI5* are positive regulators of ABA signal transduction. *Em1* and *Em6* are downstream response genes that are induced by ABA during seed development and germination, and their expressions are directly regulated by the transcription factor ABI5 [9,10]. High salt stress, drought stress, and ABA strongly induced the expressions of *RD29A* and *RD29B*. As shown in Figure 2, the transcripts of *ABF1*, *ABF2*, *ABF3*, *ABF4*, and *MYC2* were not substantially altered among the different plant samples. However, the expressions of *ABI3*, *ABI4*, *ABI5*, *EM1*, *EM6*, *RD29A*, and *RD29B* were remarkably upregulated in the *35S::HAOFP1* OE line compared with those of the controls. Two early seed-maturity-stage genes (*Em1* and *Em6*) displayed substantial increases, over 80- and 30-fold, respectively. In terms of *Atofp1* mutants, the gene expressions of *ABI3*, *ABI5*, *EM1*, and *EM6* were decreased to a certain degree that ranged from 0.42 to 0.64 compared to that of that of Col-0.

According to these results, AtOFP1 may serve as a regulator of ABA signaling through the positive mediation of ABA-related genes.

### 2.3. 35S::HAOFP1 Is Likely to ABA-Hypersensitive during Seed Germination and Primary Root Growth

**Figure 3:**
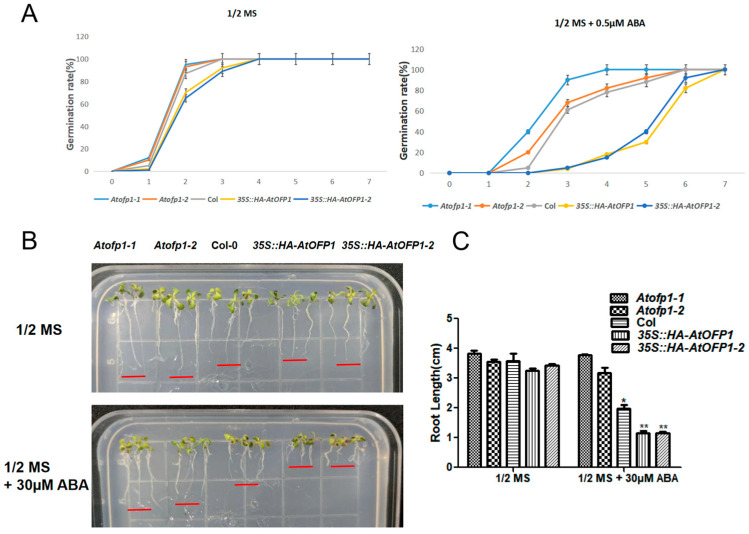
The germination rate and root elongation of Col-0, *Atofp1-1*, *Atofp1-2*, *35S::HA-AtOFP1* and *35S::HA-AtOFP1-2* under ABA treatment. (**A**) Seed germination of Col-0, *Atofp1-1*, *Atofp1-2*, *35S::HA-AtOFP1*, and *35S::HA-AtOFP1-2* after 7 days grown on medium 1/2 MS (top panel) and 1/2 MS containing 0.5 µM ABA (bottom panel). At least 50 seeds per genotype were scored in each replicate. (**B**) Root elongation of Col-0, *Atofp1-1*, *Atofp1-2*, *35S::HA-AtOFP1*, and *35S::HA-AtOFP1-2* were recorded after 7 days’ vertical cultivation on 1/2 MS without ABA (top panel) and on concentrations of 30µM ABA (bottom panel). Data shown are mean ± SD of three replicates. (**C**) Statistical analysis of root length in (**B**). Data shown are mean ± SD of three replicates. Asterisk (*) indicates statistically different from Col wild-type (*p* < 0.05), while ** represents significantly statistical difference (*p* < 0.01).

Because AtOFP1 had a positive effect on the gene expressions of *Em1* and *Em6* to verify whether AtOFP1 is involved in the ABA sensitivity to the seed germination and root growth, we planted the seeds of the *Col-0*, *Atofp1-1*, and *Atofp1-2* mutants and the *35S::HAOFP1* OE lines on 1/2 MS, with and without 0.5 μM of ABA. According to the statistical results, compared with the control group (0 μM of ABA), the seed-germination rates in the *35S::HAOFP1* transgenic lines were largely repressed under the 0.5 μM ABA treatments (Figure 3A). To be specific, under normal conditions, the germination rates of the Col-0 and mutants were 95–100% on the second day, while the germination rate was only about 70% for the *35S::HAOFP1* line. However, after 3 days of culture cultivation, all the seeds of the given samples had germinated. Moreover, after the addition of 0.5 μM of ABA, although the germination percentages of the mutants were almost the same as that of *Col-0*, the percentage was much lower in the *35S::HAOFP1* line. On the third day especially, *Atofp1-2* had a 90% seed-germination rate, Col-0 and *Atofp1-1* had about 60% seed-germination rates, and *35S::HAOFP1* had a seed-germination rate of less than 5%.

In addition, we found that the lengths of the primary roots in *35S::HAOFP1* were more strongly inhibited by ABA than those of the other samples (Figure 3B,C). After vertical culture for 7 days, the root lengths of all the lines were almost the same as on the 1/2 MS solid medium. However, on the 30 μM ABA medium, while the primary roots of the mutants were almost unaffected and continued to elongate, Col-0 inhibited the root growth by about 30%, and the inhibiting effect in the *35S::HAOFP1* OE line was almost 50%. Thus, we preliminarily concluded that AtOFP1 is involved in the regulation of the plant response to exogenous ABA during the early stage of seedling development.

### 2.4. AtOFP1 Target Genes Obtained from ChIP-SEQ Peaks Were Mainly Mitochondrial-Complex Subunits

**Figure 4:**
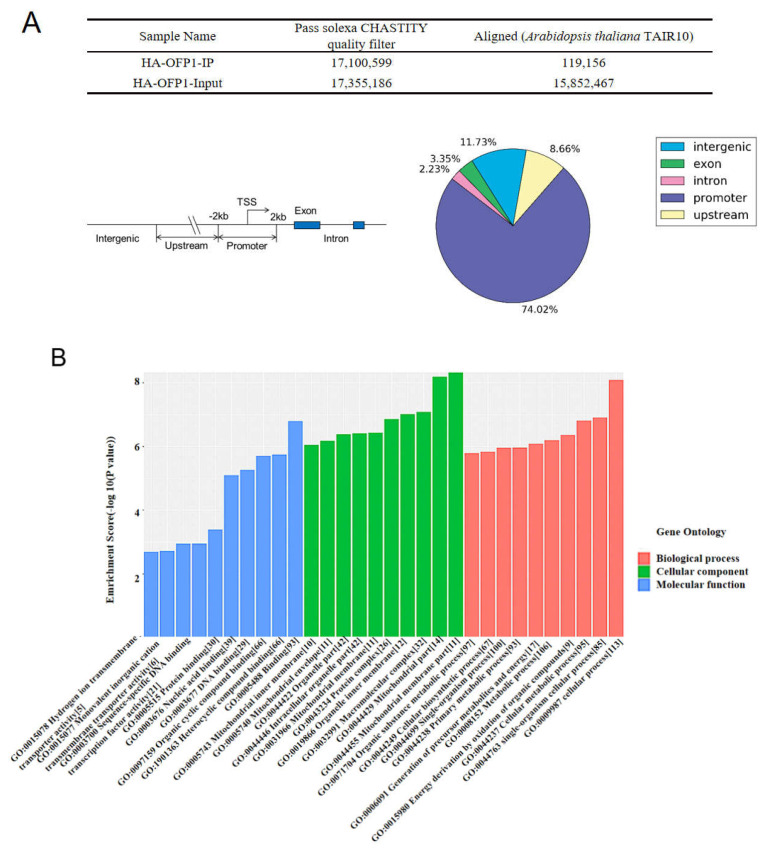

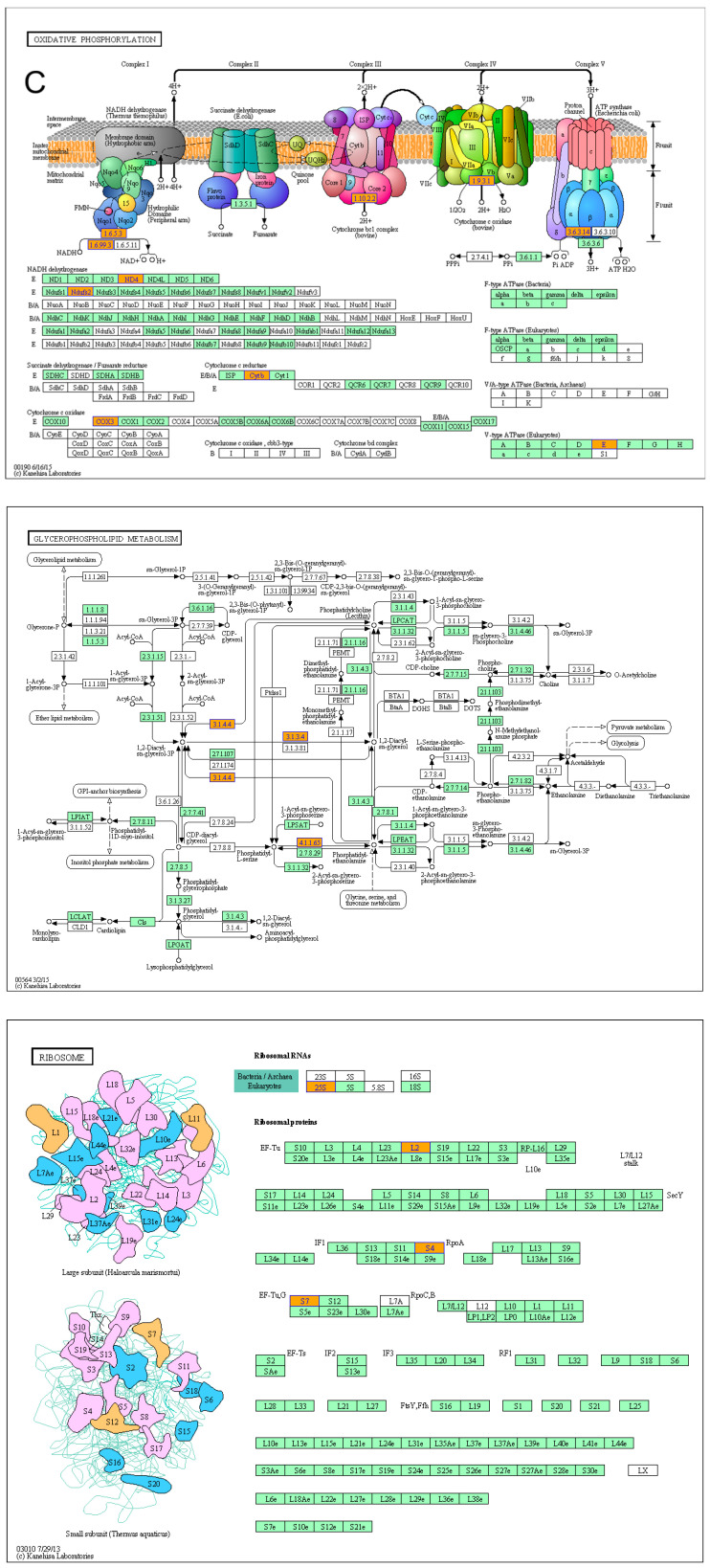
CHIP−SEQ analysis in *35S::HAOFP1* transgenic *Arabidopsis* plants. (**A**) The CHIP Peaks exploration from the comparison of *35S::HAOFP1* and *Arabidopsis* reference genome. The number of pass-filtering reads and uniquely aligned reads are listed in the table (Top). Among them, Promoters Peaks were defined as 2000 bp upstream and downstream from the TSS (Transcriptional Start Site). Upstream Peaks were seen as >2000 bp upstream to a maximum of 20,000 bp upstream from the TSS. Intron Peaks and Exons were defined the same as that of UCSC RefSeq. Intergenic Peaks were regarded as the other genomic regions not included in the above four regions. (**B**) Histogram of Gene Ontology (GO) Classification. The *x*-axis represents the proportion of the annotated genes within three classification systems including BP (Biologically Process), CC (Cellular Component), and MF (Molecular Function). The *y*-axis represents the enrichment score values of the top ten most significant enrichment terms. Different colors showed the distinguished enrichment groups. (**C**) KEGG pathway enrichment from CHIP−SEQ. KEGG determine the biological pathways that are significant enrichment of differentially expressed mRNAs. Oxidative phosphorylation (ath00190), Ribosome (ath03010), and Glycerophospholipid metabolism (ath00564) were the top three significant clusters; *p*-value cut-off is 0.05.

Some transcription factors typically function by binding to the DNA-regulatory sequences to regulate their downstream target genes [32]. To ascertain that the genes that are potentially mediated by AtOFP1, we performed a ChIP assay on the 10-day-old *35S::HAOFP1* transgenic *Arabidopsis* seedlings that expressed an AtOFP1–HA fusion protein and utilized an anti-HA tag antibody. We used Illumina HiSeq 2000 (Illumina, San Diego, CA, USA) to sequence the immunoprecipitated DNA average fragment size of the input and anti-HA ChIP libraries from 371 to 400 bp. After passing the quality filter using Solexa CHASTITY, we employed BOWTIE software (V2.1.0) to align the obtained clean reads with the *Arabidopsis* genome. Figure 4A lists the numbers of reads that passed the quality filter and aligned reads in the input and OFP1-IP groups. We then employed Model-based Analysis of ChIPSeq (MACS2) software to search the enriched regions. A *p*-value of <10^−3^ indicated that the enrichment region was a peak that reflected the best binding site of the AtOFP1 transcription factor. According to the results, we manually divided all the peaks into five regions. Among the 421 peaks collected, except for the 11.73% of peaks that were located in the intergenic region, the others were spread among the genic regions, which are located from 2 kb upstream of the transcription start to 2 kb downstream of the stop codon. A total of 74.02% are distributed in the promoter region, 3.35% in the exon region, and 2.23% in the intron region. In addition, 8.66% of the peaks are scattered within the upstream region.

To link the possible biological mechanisms between the transcription factor AtOFP1 and its target genes, we analyzed the enrichment of the Gene Ontology (GO) and Kyoto Encyclopedia of Genes and Genomes (KEGG) categories in this study (Figure 4B,C). GO analysis contains three layers in its classification system: biological process (BP), cellular component (CC), and molecular function (MF). In terms of the biological process (BP), most of the genes (113) are enriched in the cellular process (GO:0009987), and 106 genes are involved in metabolism (GO:0008152), including synthesis and decomposition, such as DNA replication, protein synthesis, and degradation, etc., (Table S1). A total of 35 genes participate in the response to the stimulus signal (GO:0050896). Moreover, in terms of the molecular function, these genes mainly function in binding (GO:0005488) and heterocyclic-compound binding (GO:1901363) (Table S2). The mitochondrial membrane (GO:0044455) and mitochondrial (GO:0044429) parts are primarily in the cellular component, and we predicted mitochondria to be the main distribution locations of target genes (Table S3). In terms of the KEGG annotation analysis of AtOFP1 target genes, we found three pathways clustered in the given samples: oxidative phosphorylation (ath00190), ribosome (ath03010), and glycerophospholipid (ath00564) metabolism (Table S4).

### 2.5. AtOFP1 Regulates Mitochondrial mRNA Splicing of NAD4 and NAD7

**Figure 5:**
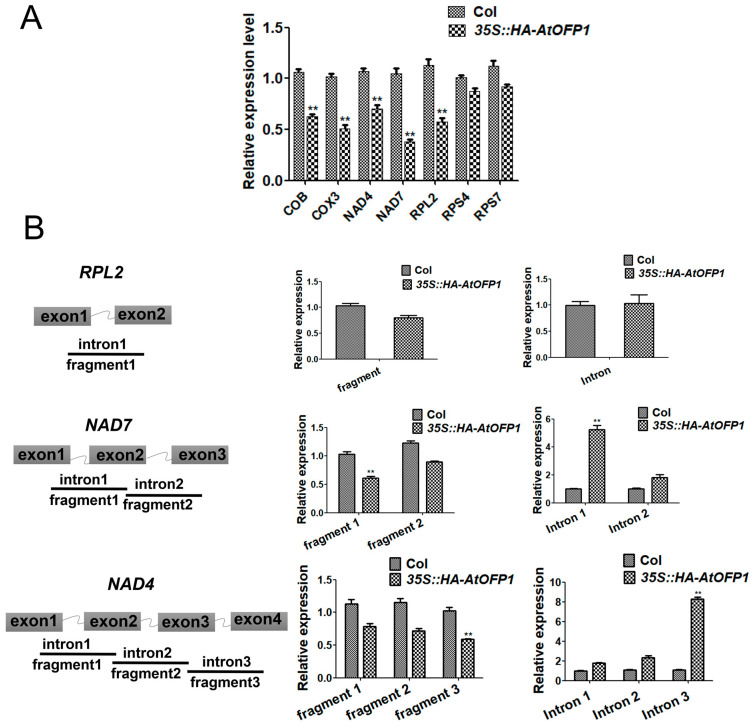
The regulation of AtOFP1 on *NAD4* and *NAD7* genes. (**A**) The expression pattern of mitochondria mature transcripts *COB*, *COX3*, *NAD4*, *NAD7*, *RPL2, RPS4*, and *RPS7* by qRT-PCR. (**B**) Splicing efficiency analysis of mitochondrial membrane genes. The different fragments of the intron-containing *NAD4*, *NAD7*, and *RPL2* transcripts were detected. Pairs of primers covering different exons or introns was used for qRT-PCR. Three independent experiments were done with three biological repeats. Values are means ± SE from one experiment. ** *p* < 0.01.

We chose *NAD4*, *NAD7*, *RPL2*, *RPS4*, *RPS7*, *COB*, and *COX3* for RT-qPCR analysis to confirm the mitochondrial-related electron transport chain (ETC)-complex subunits modulated by AtOFP1. According to the results, compared with Col-0, the mRNAs of *NAD4*, *NAD7*, *RPL2*, *COB*, and *COX3* in the *35S::HA-AtOFP1* OE line are downregulated, and we found no significant difference in the transcripts of *RPS4* and *RPS7* (Figure 5A). According to these results, AtOFP1 may affect the mRNA formation of mitochondrial ETC-complex subunits at either the transcriptional or post-transcriptional level.

Most of the mitochondrial genes contain multiple introns within the coding regions [19]. Therefore, we proposed that the normalized transcription of these genes precisely requires the post-transcriptional process. To further explore whether the reduction in the mature transcripts of these ETC-complex subunits in the *35S::AtOFP1* OE line are caused by the defect in pre-mRNA splicing, we designed specific primers inside the exons that covered different introns (three introns of *NAD4*, two introns of *NAD7*, and one intron of *RPL2*) to distinguish which intron is affected by AtOFP1. As is shown in Figure 5B, we found no significant difference in the splicing of the *RPL2* transcripts. However, compared with *Col-0*, fragment 3 (covering exons 3 and 4) in *NAD4* is smaller, but that in intron 3 (primers inside introns) is substantially larger (over sixfold) in the *35S::AtOFP1* OE line. In terms of the ETC complex I subunit *NAD7* gene, fragment 1 (covering exons 1 and 2) is smaller, but that in intron 1 (primers inside introns) is remarkably larger (more than eightfold). According to these results, the overexpression of AtOFP1 tends to disturb the splicing of *NAD4* and *NAD7*.

### 2.6. 35S::HAOFP1 OE Line Accumulates More ROS in Root Tips

**Figure 6:**
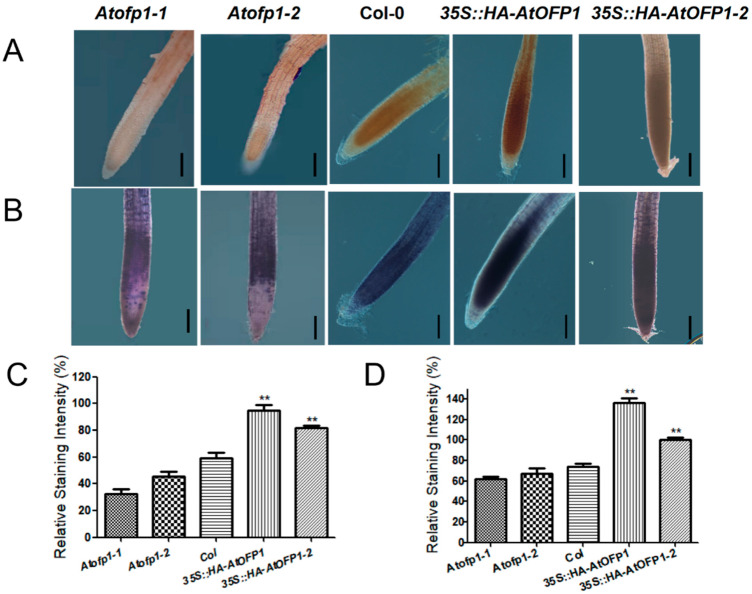
*35S::AtOFP1* OE line accumulates more ROS than the Col-0 in root tips. (**A**,**C**) represent DAB staining for H_2_O_2_ in primary root rips of the 10-day-old *Atofp1-1*, *Atofp1-2*, *Col-0*, *35S::AtOFP1*, and *35S::HA-AtOFP1-2* lines. (**B**,**D**) NBT staining for superoxide in primary root rips of the 10-day-old *Atofp1-1*, *Atofp1-2*, *Col-0*, *35S::AtOFP1*, and *35S::HA-AtOFP1-2* lines. Bars = 50 μm. DAB and NBT staining intensity were determined with Adobe Photoshop CC2019 (**C**,**D**). Asterisk (**) indicates significantly different from Col wild-type (*p* < 0.01).

The disorder of the mitochondrial ETC accumulates excess ROS and breaks the redox imbalance [17]. Because both *NAD4* and *NAD7* are encoded proteins that consist of ETC complex I in the mitochondrial genome, we introduced 3,3N-diaminobenzidine tertrahydrochloride (DAB) and nitrotetrazolium blue chloride (NBT) to detect the H_2_O_2_ and O_2_^−^ contents in the root tips, respectively, to monitor whether the overexpression of AtOFP1 further influences the change in the ROS content. According to the staining intensity of both DAB and NBT, the ROS accumulation levels in the *35S::AtOFP1* OE lines were remarkably higher than those in the wild type (Figure 6).

### 2.7. Elevated ROS Levels in 35S::HAOFP1 OE Line Cause ABA-Hypersensitive Phenotype and Are Partially Restored by Additional GSH

**Figure 7:**
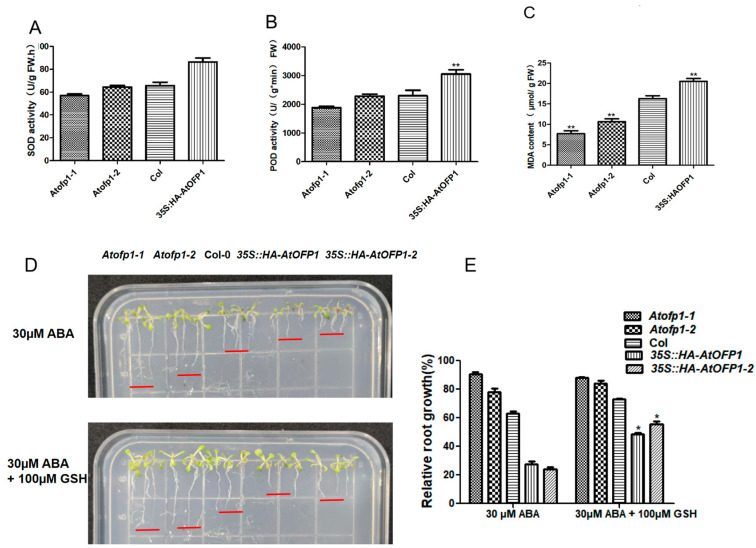
Comparison of antioxidant enzyme activities (SOD and POD) and Malondialdehyde (MDA) in the Col-0, *Atofp1-1*, *Atofp1-2*, and *35S::HAOFP1* OE lines. (**A**) The enzyme activities of SOD. (**B**) The enzyme activities of POD. (**C**) The contents of MDA. (**D**) Root elongation of *Col-0*, *Atofp1-1*, *Atofp1-2*, *35S::HA-AtOFP1*, and *35S::HA-AtOFP1-2* after 7 days on 30 µM ABA (top panel) and 100 μm GSH containing 30 µM ABA (bottom panel). (**E**) Statistical analysis of root growth in (**D**). Data shown are mean ± SD of three replicates. Asterisk (*) indicates statistically different from Col wild-type (*p* < 0.05), while ** represents significantly statistical difference (*p* < 0.01).

To the damage caused by the accumulation of ROS, plants have evolved a series of self-protection systems, including the production of antioxidant enzymes or biological macromolecules, to maintain ROS homeostasis. As shown in Figure 7A, the enzyme activity of SOD in the *35S::HAOFP1* OE line was 30% higher than that of *Col-0*, and approximately 42% higher than that of the *Atofp1-1* and *Atofp1-2* mutants. Consistent with this, the change trend of POD was similar to that of SOD, and the *35S::HAOFP1* OE line had the highest POD activity (Figure 7B). In addition, the *Atofp1-1* and *Atofp1-2* mutants had the lowest MDA contents, whereas the *35S::HAOFP1* OE line had the highest MDA content (Figure 7C). These results agree with the staining results and indicate that the overexpression of AtOFP1 requires the production of more antioxidant enzymes to resist excessive ROS production.

In previous studies, the accumulation of ROS in plants appeared to be more sensitive to ABA. To further test whether the ABA-sensitive phenotype in *35S::HAOFP1* is caused by excessive ROS, we added the reducing agent GSH for observation. As shown in Figure 7D,E, when we added an extra 100 µM of GSH to the medium containing ABA, we partially rescued the ABA hypersensitivity of *35S::HAOFP1*, which implies that the accumulation of ROS caused the sensitivity due to AtOFP1 during the seedling stage.

## 3. Discussion

The protein-interaction network can be effectively used to assess the potential functions of unknown proteins [33]. OFPs usually perform their biological functions through interactions with other proteins. *Chrysanthemum morifolium* is a model plant for understanding the regulation of flower development. More recently, according to a transcriptome sequencing and weighted correlation network analysis (WGCNA) on the variety ‘Jinba’, CmOFP is involved in the *CYC2* gene network, which plays an important role in the determination of bilateral petal symmetry. According to the Y2H and BIFC, CmOFP can physically interact with CmCYC2d [34]. Moreover, AtOFP2 and AtOFP5 regulate the orientation of the microtubule by directly interacting with the microtubule regulatory protein TONNEAU2 (TON2) [35]. In *Arabidopsis*, although we preliminarily identified 13 transcription factors in the TALE class that interact with AtOFP1, few TALE–AtOFP1 proteins function as heterodimers, according to large-scale yeast two-hybrid screening. AtKNAT3 belongs to the TALE subfamily (KNOX class II) and likely forms a positive feedback loop with ABA treatment. The *Atknat3* mutants seemed to be more insensitive to ABA than the wild type in regulating seed germination and post-germination growth [31]. In this study, we found a putative function of AtOFP1 in the ABA signaling pathway. To support this idea, we conducted both a Y2H and BiFC to illustrate the physical-interaction relationship between AtOFP1 and AtKNAT3 (Figure 1). According to the transcription analysis, the expressions of ABA-signal marker genes of *Em1* and *Em6*, as well as *RD29A* and *RD29B*, were substantially increased in the *35S::HAOFP1* OE line (Figure 2). In addition, under ABA treatment, the germination rate and root growth were both substantially inhibited in the *35S::HAOFP1* OE line, which indicates that AtOFP1 affects the hypersensitivity to ABA in the process of seed germination and seedling growth (Figure 3). According to the above data on the protein interaction, gene transcription level, and plant phenotype, AtOFP1 might work together with AtKNAT3 to respond to the ABA signaling pathway, which lays a foundation for further exploration of the mechanisms of action of AtOFP1 in plant growth and development.

Because of the curled leaves, later flowering, and reduced fertilization in the *35S::HAOFP1* line [24], in addition to protein-interaction analysis, we also used the ChIP-SEQ method to explore the AtOFP1-regulated target genes related to these phenotypes. ChIP-SEQ is a valuable tool for identifying the DNA-binding sites of transcription factors, and a novel and efficient method for selecting the potential targeted genes of TFs [36,37]. In this study, through a peak-region scan and sequence alignment with the *Arabidopsis* genome, we mined 421 genes and defined them as AtOFP1-regulated candidate target genes. According to the GO and KEGG annotations, the enriched peaks are mainly mitochondria-located genes that are related to oxidative phosphorylation (Figure 4). Mitochondria are the main productive sites in plant cells, and they tend to release energy through oxidative phosphorylation to drive the synthesis of ATP, which is a process that occurs in the inner membrane of mitochondria through a range of complexes in the electron transport chain (ETC). These complex proteins are composed of ETCs and are characterized by multiple introns. Therefore, abnormal RNA splicing damages the morphological structure and function of the ETC, which results in the distortion of the plant growth and development, including plant dwarfing, leaf curl, cotyledon deformation, flowering delay, etc. For instance, the gene loss of the mitochondrion-localized P-type PPR protein PPR18 in maize could lead to an empty pericarp phenotype, which is caused by the splicing of *NAD4* intron 1 [38]. The splicing defects of *NAD7* introns 2 impair the Complex I activity and cause growth retardation in slow-growth (*slo3*) mutants [39]. Because of the similar phenotype shared with *35S::HA-ATOFP1*, we speculated that the defect splicing of *NAD4* and *NAD7* was partially responsible for the given phenotype. Except for *NAD4* and *NAD7*, the expression levels of the *COB*-gene-encoding ETC Complex III and *COX3* gene in Complex IV were also substantially reduced in the *35S::HA-ATOFP1* line. We require further exploration as to whether AtOFP1 also participates in the mRNA processing of these genes.

Plants integrate various signaling pathways to balance their growth and stress responses. According to several studies, a tandem relationship may exist between ABA and ROS signaling [34]; that is, changes in the ROS content may be able to affect the biosynthesis and signal transduction of ABA in plants, and ABA may, in turn, regulate the expression of ROS-production and ROS-scavenging genes. For example, the ABO8 protein is a mitochondrial P-type PPR protein that is responsible for the splicing of *NAD4* intron 3. In *Arabidopsis*, the *abo8* mutant accumulates more ROS than the wild type and shows greater sensitivity to ABA treatment during seed germination and root growth. However, when we added the reductant GSH, ABA sensitivity was restored, which demonstrates that a splicing deficiency of *NAD4* in *abo8* is the cause of ABA hypersensitivity to seed germination and root growth [40]. In *Oryza sativa*, the overexpression of *OsMADS25* considerably enhanced the salt tolerance and oxidative stress by increasing the antioxidant enzyme activity (CAT) and the osmotic protective solute (MDA and proline). The expressions of ABA-dependent stress-responsive genes are also substantially elevated in overexpression plants under salinity stress and increase the root sensitivity to exogenous ABA [41]. The sensitivity of *35S::HA-AtOFP1* to ABA may be related to ROS accumulation, which confirms that AtOFP1 is involved in the crosstalk between the ABA and ROS signals.

## 4. Materials and Methods

### 4.1. Plant Materials and Growth Conditions

The *Arabidopsis thaliana* ecotype Columbia (Col-0), two lines of mutant *Atofp1-1*, *Atofp1-2*, and *35S::HAOFP1* transgenic plants (OE line) were launched to this study. All the seeds were sterilized by 75% ethanol (2 min) and subsequently with 5% NaClO (8 min), followed by ddH_2_O (five times). Then, they were placed on a 0.8% agar medium of 1/2 Murashige and Skoog (MS medium, pH 5.7) in darkness for 48 h. After stratification, the plates were put into the growth room (22 °C) with 6 h/8 h (light/dark) long-day photoperiodic conditions.

### 4.2. RNA Extraction and Gene Expression Profile Analysis

Total RNA of Col-0, *Atofp1-1*, *Atofp1-2*, and *35S::HAOFP1* seedlings was isolated from 10-day-old *Arabidopsis* using EasyPure^®^ RNA Kit (TransGen Biotech, Beijing, China). Then, the first-strand cDNA was synthesized with the HiScript III 1st Strand cDNA Synthesis Kit (Vazyme Biotech, Nanjing, China) based on the manufacturer’s instructions. Then, the qRT-PCR reaction was performed with an AceQ Universal SYBR qPCR Master Mix (Vazyme Biotech, Nanjing, China) with the gene-specific primers listed in Table S5. The program was set on a 96-well System (Axygen, San Francisco, CA, USA), and the cycling conditions were as follows: initial denaturation of 5 min at 95 °C, followed by 10 s at 95 °C for denaturation, 30 s at 60 °C for annealing, and 30 s at 72 °C for extension (40 cycles). Then, for the melting curve, 95 °C for 15 s, 60 °C for 1 min, 95 °C for 15 s. The relative expression levels of genes were normalized by Actin2/8 and calculated using the 2^−^^ΔΔCT^ method.

### 4.3. Plasmid Constrction and Protein-Protein Interaction Assays

Yeast AH109 competent cells were prepared by PEG/LiAc method according to the operation procedure of Matchmaker^TM^ Gold yeast transformation system (Clontech, Mountain View, CA, USA). In this system, the AtOFP1 coding sequence was cloned into the pGBKT7 vector containing the GAL4-binding domain (BD), while full-length AtKNAT3 or truncated fragments including KNOX1, KNOX2, MEINOX, and HD domain were amplified by PCR and inserted into the NdeI and BamHI sites of pGADT7 vector with GAL4 activation domain (AD). Then, the pGADT7–AtKNAT3 and pGBKT7–AtOFP1 were co-transferred into AH109 yeast strain. At the same time, empty vector (BD) with pGADT7–AtKNAT3 served as negative control, and pGBKT7–AtOFP1 + AD–EMPTY group was detected to exclude self-activation. The recombinant vectors were selected on synthetic dropout (SD) medium without tryptophan (Trp) and leucine (Leu) (SD/-Trp/-Leu) or SD medium without Trp, Leu, histidine (His), and adenine (Ade).

For BIFC assays, full-length cDNAs of AtOFP1 and AtKNAT3 were cloned into pENTR Gateway entry vector (pENTR-D-TOPO, Invitrogen) followed by their respective insertion into GFP-fused destination vectors. Then, the GFPC–AtKNAT3 and GFPN–AtOFP1 were co-transformed into *Arabidopsis* protoplasts to observe the GFP fluorescence. Images was visualized with a confocal laser scanning microscope.

### 4.4. ABA Treatment, Seed Germination and Root Length Measurement

For germination rate, 50 seeds were planted in each plate containing different concentrations of ABA or GSH and cultured in the same environment. The seed germination was observed and counted every day. For the root growth experiment, after the seedlings were grown for 2 weeks and until the root tips were at the same level, the seedlings were carefully moved with sterile bent hook spicules to 1/2 MS medium with/without 30 µM ABA or 100 µM GSH chemicals. The petri dishes were placed vertically into the light incubator. After 5 days, photos were taken, and the root length was measured with ImageJ software (version 1.8; National Institutes of Health, Bethesda, MD, USA).

### 4.5. CHIP-SEQ

This experiment was completed by KangChen Bio-Tech Company (Shanghai, CHINA). CHIP Assay was performed by Quant-It DSDNA Assay Kit (Q33120). For tissue collection and fixation, 2 g of two-week-old *35S::HAOFP1* seedlings was put into 37 mL of cross-linking buffer, and after 10 min vacuum, 2.5 mL 2M glycine was added to terminate the crosslink reaction by additional vacuuming for 5 min. After three washes with cold ddH_2_O, samples were dried with blotting paper and milled to fine powder with liquid nitrogen. Then, the nuclei were suspended for sonication with 65% power (30 s on, 30 s off) to shear the DNA to an average size of 0.3–1 kb. After 4 °C centrifuge at 12,000× *g* for 5 min, the supernatant was transferred to a new 2 mL centrifuge tube, and 120 μL was taken as input DNA.

The pretreated chromatin was added with protein ien agarose beads (25% suspension) for pre-clearing. After being incubated at 4 °C for 50 min in a shaking table, each sample was centrifuged at 16,000× *g* for 10 min, and the supernatants were divided equally into two samples. One sample received 3 μL of an anti-HA tag antibody (Abcam, ChIP Grade, ab9110) as the IP group, and the other as negative control. The purified antibody was added with 3 μL and cultured at 4 °C for 1 h in a shaking table.

Subsequently, beads were washed five times with IP buffer (20% SDS, 0.5 M EDTA pH = 8.0, 1 M Tris-HCl pH = 7.8, 50× cocktail), and samples were pelleted by centrifuge at 3800× *g* for 2 min. Then, 100 μL pre-cold elution buffer (10% Triton X-100, 0.5 M EDTA pH = 8.0, 5 M NaCl, 1 M Tris-HCl pH = 7.8, 50× cocktail) was added to remove the protein–DNA complex from the beads. After incubation in shaker at 37 °C for 1 min, spinning at speed of 3800× *g* for 1 min, the supernatant was transferred to a new centrifuge tube; this step was repeated twice. For cross-linking the samples, 10 μL of 0.5 M EDTA, 20 μL of 1 M Tris-HCl (pH 6.8), 1 μL of 1 mg/mL RNase A, and 1 μL of 20 mg/mL Proteinase K were mixed into each tube at 45 °C for 1 h. For DNA purification, the precipitated DNA was added with 2.5 times volume of ethanol, 1/10 volume of 3 M NaAc (pH = 5.4), and 1 μL glycogen overnight. DNA was recovered into 50 μL EB using QIAGEN PCR recovery kit.

To prepare 10ng DNA samples for Illumina sequencing, a dA base was added to the 3’ end of each chain by Klenow polymerase, and Illumina’s genomic adapters were linked to DNA fragments. After PCR amplification, the enrichment products of ~200–500 bp were separated from the gel and purified by Qiaquick Gel Extraction Kit. The completed library was quantified with Agilent 2100 bioanalyzer. After the sequenced reads were generated by the Illumina HiSeq 2000, the clean reads were aligned to *Arabidopsis* genome (UCSC TAIR10) by BOWTIE software (V2.1.0). Additionally, MACS v1.4.2 (Model-based Analysis of ChIP-seq) software was used to detect the peak from ChIP-seq data, followed with peaks annotation by UCSC RefSeq database. The GO categories were derived from Gene Ontology (www.geneontology.org, accessed on 3 December 2020), while KEGG was obtained from Kyoto Encyclopedia of Genes and Genomes database; *p*-value ≤ 0.05 is recommended for significance [42].

### 4.6. Analysis of Root-Tip ROS Staining

For H_2_O_2_ content detection, the roots of Col-0 and *35S::HA-AtOFP1* OE seedlings were placed in 50 mM Tris-HCl containing 0.1 mg/mL DAB (3′,3′-diaminobenzidine, pH = 5.0) and cultured under dark for 10 h.

For the measurement of superoxides, the given samples were immersed in 20 mM potassium phosphate buffer containing 0.1 M NaCl and 2 mM NBT (nitroblue tetrazolium containing, pH = 6.1). After 3 h incubation under dark condition, the seedlings were rinsed three times with ddH_2_O, and the dyed seedlings were section-prepared and observed under phase contrast microscope and photographed.

### 4.7. Measurement of Enzymatic Activity (SOD and POD) and Estimation of Malondialdehyde (MDA)

The seedlings of wild-type Col-0, *Atofp1-1*, *Atofp1-2* mutants, and *35S::HA-AtOFP1* OE line were weighed and placed into a mortar, and 10 mL buffer solution (0.05mol/L dipotassium phosphate buffer solution, PH = 7.8) was added to samples to ground into homogenate, then centrifuged at 4000× *g* rpm for 10 min at room temperature. The prepared enzyme solution was further utilized to measure the activity of SOD, POD, and the content of MDA according to the method previously described [43].

### 4.8. Data Statistical Analysis

SPSS software was used for all statistical data analysis, and Student’s *t*-test was applied for the significant difference judgement between the indicated groups and control. GraphPad Prism 5 (GraphPad Software, Inc., La Jolla, CA, USA) was used to plot the results. All experiments results presented in the Figures were in the form of “mean ± standard deviation”. ** was significant analysis between data (*p* < 0.01).

## 5. Conclusions

In this study, we obtained overall insight into the transcriptional factor AtOFP1, both in protein–protein and protein–DNA interactions. On the one hand, we found that AtOFP1 is involved in the ABA signaling pathway through the AtOFP1–AtKNAT3 complex. On the other hand, by using ChIP-SEQ to explore AtOFP1-mediated candidate target genes at the genome-wide level of *Arabidopsis*, we proved that AtOFP1 tends to facilitate intracellular ROS accumulation. By screening AtOFP1 target genes and interacting proteins, we mainly focused on the intersection of various stress responses from the perspective of signal correlation. We hope to obtain more critical regulatory factors that can improve the comprehensive stress resistance and be applied to the innovation and improvement of plant germplasm resources.

## Data Availability

Not applicable.

## Supplementary Materials

The following supporting information can be downloaded at: https://www.mdpi.com/article/10.3390/ijms23137427/s1.

## Abbreviations

At	*Arabidopsis thaliana*
OFP	Ovate Family Protein
Col-0	Columbia-0
qRT-PCR	Real-Time Quantitative Reverse Transcription PCR
ABA	Abscisic acid
ABREs	ABA-responsive elements
Em1	Embryogenesis abundant 1
RD29A	Responsive to desiccation 29A
ETC	Electron transfer chain
GO	Gene ontology
KEGG	Kyoto Encyclopedia of Genes and Genomes
DAB	3,3N-Diaminobenzidine Tertrahydrochloride
NBT	Nitrotetrazolium Blue chloride
SOD	Superoxide dismutase
POD	Peroxidase
MDA	Malondialdehyde
