# Supplementary material for: Transcription Factor AtOFP1 Involved in ABA-Mediated Seed Germination and Root Growth through Modulation of ROS Homeostasis in Arabidopsis
Source: Int J Mol Sci. 2022 Jul 4;23(13):7427. doi: 10.3390/ijms23137427 (PMC9267126; doi:10.3390/ijms23137427)
Supplement: Supplementary file 1 [file ijms-23-07427-s001.zip › ijms-1753068-supplementary/Supplementary Files/Table S5.pdf]

Clone Primers used in this study

Primer Name	Sequence
AtOFP1-BD-F	CATGGAGGCCAGTGAATTCATGGGTAATAACTATCGG
AtOFP1-BD-R	TGCAGGTCGACGGATCCTTTGGAATGGGG
AtKNAT3-AD-F	CATATGGCCATGGAGGCCGAATTCATGGCGTTTCATCAC
AtKNAT3-AD-R	TCGAGCTCGATGGATCCCCTACGCGAACCGCTCTCT
AtKNAT3-GFP-F	actagtaagcttcggatccATGGCGTTTCATCAC
AtKNAT3-GFP-R	cttgctcaCcatggatcCCTACGCGAACCGCTCTCT
AtOFP1-GFP-F	cgactgcagatctagagctcATGGGTAATAACTATCGG
AtOFP1-GFP-R	ttctgcttgtcggcgagctcTTTGGAATGGGG

Primers for Quantitative Real-Time PCR

Primer Name	Sequence
ABF1-qF	TGCAGAAGAAACAGGCTGAA
ABF1-qR	GACCGGTAAGGGTTCTTCTCA
ABF2-qF	TTGACTCTGCCTCGAACGCTTAG
ABF2-qR	CCTCTGACTCTGACTCTGACTCTG
ABF3-qF	CGTTCTCAACCTGCAACACA
ABF3-qR	TCATAGGATGGTTATGAATTCCAAG
ABF4-qF	CGTTGACGTTGCCTAGAACAATTAG
ABF4-qR	GTTATCTTCTCTTACAACCCCAGC
MYC2-qF	CGCTGTTGATGAGGAGGTG
MYC2-qR	TCCCAAACACTCCTCCTT
ABI3-qF	CGCATCAGGAAACTGTGACG
ABI3-qR	CGTGTCGTCGTCTTGGCTAA
ABI4-qF	AAGTGAGTGAGAAGAGAGTGTAAGT
ABI4-qR	ACCGTAATCTCTTTTACGAATTCC
ABI5-qF	TGGTGAAGGCTGGTGTGGT
ABI5-qR	TGATGGGTCACCCACACCC
Em1-qF	CGAGCTACTAGTGTCCGCTGCA
Em1-qR	GTAAAACCAACCGGCAACCGCA
Em6-qF	CTCAACAAGAGAAGAAGCAGCTGG
Em6-qR	GGTCTTGGTCCTGAATTTGGATT
RD29A-qF	GGCGTAACAGGTAAACCTAGAG
RD29A-qR	TCCGATGTAAACGTCGTCC
RD29B-qF	TCATGAGAAGGGAGCATCCAA
RD29B-qR	TCCTCCGATGCCGGAACATT
ACTIN-qF	CAAGGCCGAGTATGATGAGG
ACTIN-qR	GAAACGCAGACGTAAGTAAAAAC
cob	TGCCGGAATGGTATTTCCTA/GCCAAAAGCAACCAAAACAT
cox3	CCGTAACTTGGGCTCATCAT/AAACCATGAAAGCCTGTTGC
nad4	AATACCCATGTTTCCCGAAG/TGCTACCTCCAATTCCCTGT
nad7	ACTGTCACTGCACAGCAAGC/CATTGCACAATGATCCGAAG
rpl2	CCGAAGACGGATCAAGGTAA/CGCAATTCATCACCATTTTG
rps4	ACCCATCACAGAGATGCACA/TCACACAAACCCTTCGATGA
rps7	CTCGAACTGAACGCGATGTA/AAGCTGCTTCAAGGATCCAA
nad4 exon1-2	ATTCTATGTTTTTCCCGAAAGC/GAAAAACTGATATGCTGCCTTG
nad4 intron1 exon2	CCGTATGATGCGGAAGTCTC/GAAAAACTGATATGCTGCCTTG
nad4 exon2-3	AATACCCATGTTTCCCGAAG/TGCTACCTCCAATTCCCTGT
nad4 intron2 exon3	AATACCCATGTTTCCCGAAG/TGCTACCTCCAATTCCCTGT
nad4 exon3-4	TTCCTCCATAAATTCTCCGATT/TGAAATTTGCCATGTTGCAC
nad4 intron3 exon4	TCTAGCTTGGTTCGGAGAGC/TGAAATTTGCCATGTTGCAC
nad7 exon1-2	ACCTCAACATCCTGCTGCTC/AAGGTAAAGCTTGAAGATAAGTTTTGT
nad7 intron1 exon2	ACGGTTTTTAGGGGGATCTG/AAGGTAAAGCTTGAAGATAAGTTTTG

nad7 exon2-3	GAGGGACTGAGAAATTAATAGAGTACA/TGGTACCTCGCAATTCAAA
nad7 intron2 exon3	AGTGGGAGAGCCGTGTTATG/TGGTACCTCGCAATTCAAAA
nad7 exon3-4	ACTGTCACTGCACAGCAAGC/CATTGCACAATGATCCGAAG
nad7 intron3 exon4	TAAAGTGAAGTGGTGGGCCT/CATTGCACAATGATCCGAAG
nad7 exon4-5	GATCAAAGCCGATGATCGTAA/AGGTGCTTCAACTGCGGTAT
nad7 intron4 exon5	CGGCCAAATGACTACAGGAT/AGGTGCTTCAACTGCGGTAT
