# Supplementary figures and images for: Transcription Factor AtOFP1 Involved in ABA-Mediated Seed Germination and Root Growth through Modulation of ROS Homeostasis in Arabidopsis
Source: Int J Mol Sci. 2022 Jul 4;23(13):7427. doi: 10.3390/ijms23137427 (PMC9267126; doi:10.3390/ijms23137427)

## Slide 1
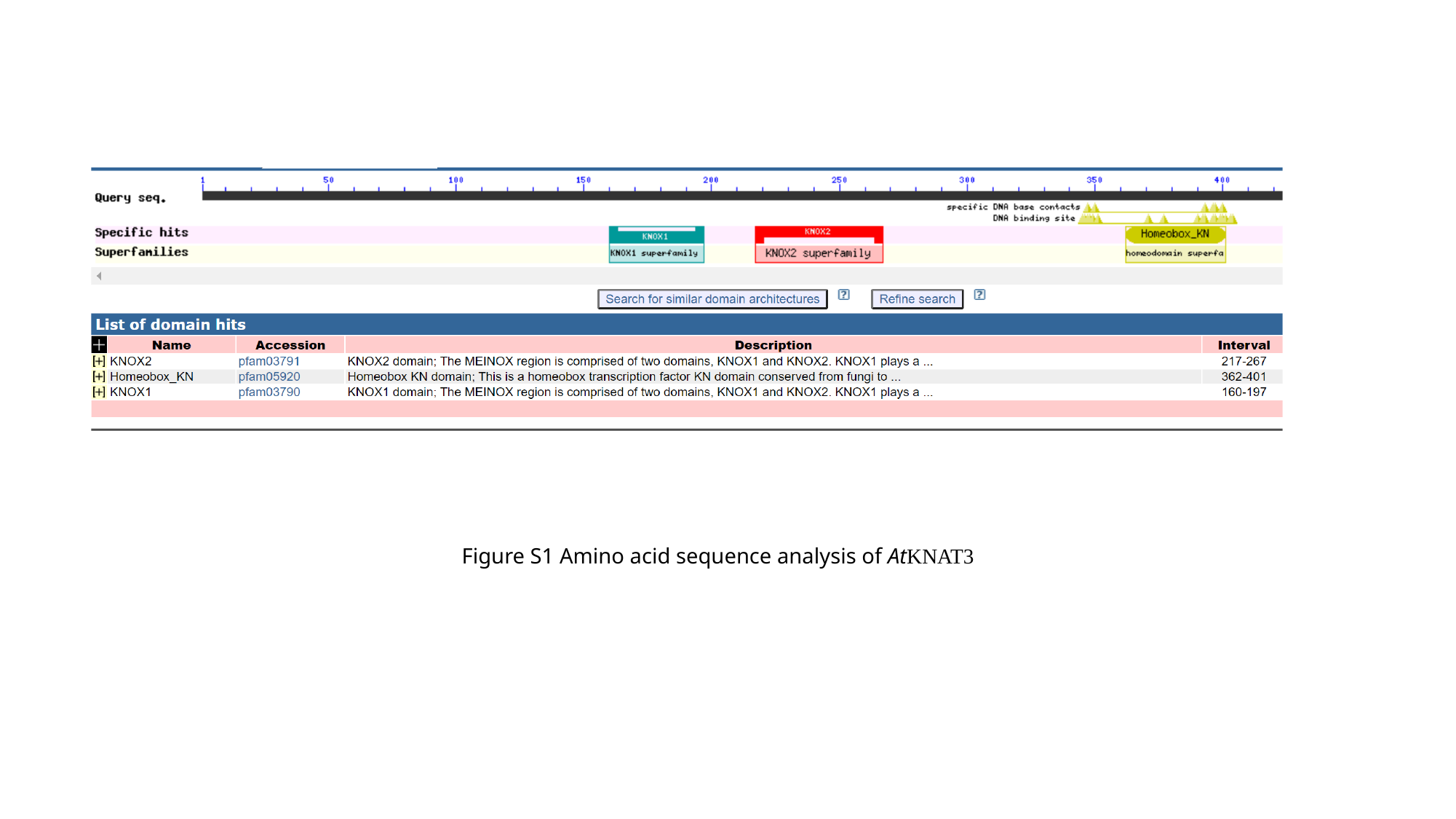

Figure S1 Amino acid sequence analysis of AtKNAT3

Supplement: Supplementary file 1 [file ijms-23-07427-s001.zip › ijms-1753068-supplementary/Supplementary Files/Figure S1.pptx]
